# Changing health behaviors using financial incentives: a review from behavioral economics

**DOI:** 10.1186/s12889-019-7407-8

**Published:** 2019-08-07

**Authors:** Ivo Vlaev, Dominic King, Ara Darzi, Paul Dolan

**Affiliations:** 1 Warwick Business School, University of Warwick, Coventry, UK; 2 Centre for Health Policy, Imperial College London, London, UK; 3 Department of Surgery and Cancer, Imperial College London, London, UK; 4 Department of Psychological and Behavioural Science, London School of Economics, London, UK

**Keywords:** Behavior change, Healthcare, Incentives, Behavioral economics, Nudge

## Abstract

**Background:**

Incentives are central to economics and are used across the public and private sectors to influence behavior. Recent interest has been shown in using financial incentives to promote desirable health behaviors and discourage unhealthy ones.

**Main text:**

If we are going to use incentive schemes to influence health behaviors, then it is important that we give them the best chance of working. Behavioral economics integrates insights from psychology with the laws of economics and provides a number of robust psychological phenomena that help to better explain human behavior. Individuals’ decisions in relation to incentives may be shaped by more subtle features – such as loss aversion, overweighting of small probabilities, hyperbolic discounting, increasing payoffs, reference points – many of which have been identified through research in behavioral economics. If incentives are shown to be a useful strategy to influence health behavior, a wider discussion will need to be had about the ethical dimensions of incentives before their wider implementation in different health programmes.

**Conclusions:**

Policy makers across the world are increasingly taking note of lessons from behavioral economics and this paper explores how key principles could help public health practitioners design effective interventions both in relation to incentive designs and more widely.

## Background

The effect of individual behavior on health outcomes is considerable with estimates that up to 40% of premature deaths in the developed world are attributable to unhealthy behaviors, such as smoking, poor diet and sedentary lifestyle [1]. Reducing morbidity and mortality losses in the future is likely to depend as much on motivating changes in behavior as on developing new treatments or technologies and many countries and health systems are now directing resources to this end [2, 3].

A range of tools are at the disposal of policy makers seeking to influence behavior including legislation, price signals (taxes and subsidies) and information campaigns. Although the use of incentives in wider public policy is nothing new, their role in encouraging health behaviors is a relatively recent phenomenon [4]. Incentives can take a number of forms including cash or alternatively vouchers that can then be exchanged for desirable items. The apparent enthusiasm for using incentives to influence health behaviors has come about as the full economic and social costs of unhealthy behaviors have become apparent and with the finding that health behaviors can be significantly affected by the structure of economic incentives that individuals face [5, 6]. Examples of incentive schemes recently implemented in the United Kingdom include the *‘Give It Up For Baby’* programme in Tayside, Scotland to encourage pregnant smokers to quit the habit and the *‘Pounds for Pounds’* scheme in Kent, England to influence weight control [7]. In diabetes care, incentive programs directed at patients show promise as a means to influence patient behavior and intermediate outcomes such as weight loss [8].

But widespread concerns do exist and tend to center on the potentially coercive impact of using incentives and the ‘unfairness’ of rewarding people for doing things that are already in their own interest [9, 10]. We share some of those reservations, but rather than adding further to this normative debate, we will focus herein on positive ways in which we might give incentives the best opportunity to work if and when they are considered appropriate.

We will look to see whether it is possible to provide further guidance on how best to configure and implement incentive schemes using evidence from behavioral economics. Behavioral economics has come to prominence following the publication of Richard Thaler and Cass Sunstein’s book *Nudge* [11], but the science underlying it is built on decades of applied research by the likes of Daniel Kahneman and Amos Tversky [12, 13]. In contrast to economic models of rational choice suggesting that we respond to information and price signals, insights from across the behavioral sciences suggest that human behavior is actually influenced greatly by the context or environment within which many of our decisions are taken. Nowadays ‘dual process’ theories can be found in social, personality, cognitive, clinical and health psychology [14]. Two general paradigms for behavior change have emerged over the years. The first type aims to change high-order cognitions such as beliefs and attitudes as a route to influencing deliberate responses by using persuasion and education campaigns. The second approach aims to influence lower-order mental processes, thus triggering spontaneous responses, by changing the context or environment within which the person acts without necessarily changing underlying higher-order cognitions such as beliefs and attitudes. In other words, the distinction is between behaviors resulting from internally cued, reflective, and intentional changes versus behaviors resulting from externally cued, automatic and (often subconscious) reactive changes [15].

Governments around the world are taking notice of the potential role behavioral economics could play in designing more effective public policy. A prominent example is the coalition Government in the United Kingdom, who have so far made recommendations on how public behavior around charitable giving and preventive health could be influenced using the Mindspace framework for behavior change [16–19]. Intelligent design of incentive schemes is one of the key tools in this new ‘behavioral’ approach.

## Financial incentives in healthcare

It is well established that a higher price reduces consumption [20]. So, we see that smoking consumption in Europe has been seen to decrease by about 5% for every 10% increase in the real price of cigarettes and that increasing the price of alcohol is among the most effective options for reducing consumption [21, 22]. Price signals to influence behavior in healthcare can take forms besides traditional ‘sin taxes’ for example by subsidising healthy behaviors or rewarding adherence to a treatment programme. The increase in the last decade of schemes aimed at changing the health-related behavior of the public has been accompanied by evidence that even small incentives can positively influence choices [23–25]. Having said that, a recent review of the evidence for the effects of economic instruments (prices or income) on dietary and physical activity behaviors and corollary outcomes [26] revealed that evidence is limited in terms of the potential for causal inference and yields ambiguous or inconsistent findings (the evidence is also mostly about impacts on diet, with very limited evidence for impacts on physical activity). Those findings highlight the need to implement robustly designed interventions and evaluations of the logic models and programme theories involved.

In clinical psychology, the contextual-change route has taken a substantial share of research, because classical behavior therapy and cognitive-behavioral therapy focus on underlying learning processes and environmental contingencies of reinforcement [27, 28]. Schedules of contingent reinforcement are also a key to the success of rewards (materialised incentives) and often used in behavioral psychology circles. For example, providing contingent rewards is used in interventions developing constructive habits. This reinforcement principle has been successfully employed to treat drug addiction and substance misuse (including smoking and alcohol consumption) and to improve medication compliance [29]. Such interventions usually include earning money or points contingent on the patients’ specific behaviors.

The effectiveness and long-term sustainability of behavioral change when incentives are targeted at the more challenging and complex behaviors such as smoking and obesity is less well known [30–33]. Also, given the opportunity costs of the changed behavior is the same across income groups, we may also expect to see that as £1 to a poor person is worth more than £1 to a rich person, small incentives are also likely to be more effective in low-income groups [34].

## A review from behavioral economics

We identified interesting examples from the literature through a search strategy of electronic databases including (PubMed, EMBASE, PsychInfo) using keywords. We reviewed Longitudinal, cross-sectional and retrospective studies of interventions using incentives to change health behaviors, including patients and/or providers of healthcare. We also included systematic reviews of such interventions. Our team independently reviewed articles and compared and discussed interpretations. Results were synthesized under thematic classification and abstraction. Because this is a debate article, we have not provided a review of all those studies. We do no aim to synthesize the evidence. Rather, in our manuscript we offer a theoretical investigation of incentive design and behavior change.

Although success has been seen with a number of financial incentive schemes targeting preventive health behaviors, some financial incentives have not worked at all well, or even at all in the case of reducing levels of obesity [33]. It may be that incentives are unlikely to work at a cost-effective level in changing certain complex behaviors. Another contributing factor could be that too little thought has been put into the design of incentive schemes previously implemented. Roland Fryer has demonstrated how important design is when thinking about how financial incentives can be used to improve educational achievement [35]. In a series of school based randomized trials, incentives were only found to be effective when they were given for inputs to the educational production function. Incentives tied to educational outputs were not effective. Qualitative data suggested that because students do not understand the education production function, they were not able to ‘turn their excitement about rewards’ into meaningful achievement. The same may be true for people offered incentives to lose weight or stop smoking.

Those choosing the format of incentive schemes will have many options available to them when thinking about design. Let us take a theoretical example of an incentive scheme to encourage participation in a weight-loss programme. The reward may be given for attending classes and it could be given at the beginning of the programme, at its completion or in increasing or decreasing increments as classes are attended. Alternatively, the incentive could be given dependent on actual weight loss targets that result from following the programme. Different scheme designs are likely to lead to different outcomes.

It is important to think about the most effective design of incentive schemes, as our responses can be shaped by a range of predictable biases and heuristics [12, 13, 36]. Behavioral economics provides us with a number of robust psychological phenomena that help explain the decisions we make in a range of settings, including savings, health and education [15, 19]. Evidence suggests that human behavior is led by our very human and fallible brain and the context or environment in which many of our choices are taken. The finding being that small changes in context (nudges) can affect behavior as much as large price changes [11, 37]. Such effects or ‘nudges’ can be applied to the design of more effective incentive schemes and include the following.

### Losses loom larger than gains

It has been demonstrated that we react more to losses than to gains of equivalent magnitude [38], which is embedded into the well-known (Nobel prize winning) Prospect Theory of risky choice. Loss aversion implies that someone who loses £10 from his or her pocket will lose more satisfaction than another person would gain satisfaction from a £10 windfall. Most incentive schemes tend to offer rewards to participants but inducing some feeling of loss if they fail to do something may be more motivating than rewarding them by the same amount. So, instead of providing a £10 reward for each of the ten sessions of a weight loss programme, it may be more effective to provide £100 at the end of the programme, with all missed sessions attracting a more salient and painful £10 loss. Using a behavioral economics framework, one randomised study has shown the short-term effectiveness of such a scheme. Individuals in this programme contributed to a matched deposit contract that rewarded them if they met or exceeded their weight loss goal but led to the loss of the reward if they failed [39]. Loss aversion is one of the most robust phenomenon from behavioral economics and could be used more widely across incentive schemes.

An interesting intervention tested the impact of financial incentives framed as a gain or loss to promote Chlamydia screening in students, mimicking the standard outreach approach to student in halls of residence [40]. This was a in a cluster randomized trial (*N* = 1060; age 18–24 years). The students were offered (depending on condition) £5 voucher vs. £200 lottery. In the control group the screening rate was 1.5%, while the lottery increased screening to 2.8% and the voucher increased screening to 22.8%. Incentives framed as gains were more effective than loss-framed incentives (10.5% vs. 7.1%, respectively). This work contributes to the literature by testing the predictive validity of Prospect Theory to change health behavior in the field.

In another domain, such a framing manipulation was used to increase factory worker productivity in a field experiment [41]. They find conditional incentives framed as both “losses” and “gains” increase productivity for both individuals and teams. In addition, teams more acutely respond to bonuses posed as losses than as comparable bonuses posed as gains. The total team productivity is enhanced by 1% purely due to the framing manipulation. Another interesting intervention tested the power of loss aversion to improve teacher performance [42]. During the 2010–11 Chicago school year teachers were randomly asked to participate in a pay-for-performance program with “gain” and “loss” treatments. The “gain” group received traditional financial incentives at the end of the year in the form of bonuses linked to student achievement. Those teachers in the “loss” group were paid a lump-sum in advance and asked to give back the money if their students did not meet performance targets. Teachers in both conditions received the same monetary bonus if they reached the same performance targets. This approach resulted in increases in math test scores for the loss condition by an equivalent of increasing teacher quality by more than one standard deviation. The gain treatment yields smaller and statistically insignificant results. The authors attribute the significant difference between the loss and gain condition to the loss aversion framing. Those intervention techniques could be used to improve the productivity of the healthcare workforce too.

### Overweighting of small probabilities

There is good evidence that people place more weight on small probabilities than standard economic theory would suggest [43], which is another essential element of Prospect Theory. This helps explain the widespread popularity of lotteries and insurance. Although the tendency to overweight the probability of unlikely but salient outcomes can lead to problem gambling [44], it can also be used to positive effect using lottery based public policy interventions. Lottery based financial incentive programs have been seen to be effective in a weight loss intervention and in improving warfarin adherence and anticoagulation control [45].

Patel et al. [46] tested the effect of different types of lottery-based financial incentives in increasing physical activity among University of Pennsylvania Employees with body mass index ≥27. All participants used smartphones to track their steps per day and received daily feedback on performance for 26 weeks (financial incentive for 13 weeks and then were follow up for 13 weeks without incentives). Daily lottery incentives were designed as a “higher frequency, smaller reward” (1 in 4 chance of winning $5), “jackpot” (1 in 400 chance of winning $500), or “combined lottery” (18% chance of $5 and 1% chance of $50). The outcome measure was the mean proportion of participants who achieved the daily goal of 7000 steps. During the intervention, only the combined lottery incentives was significantly greater than control (0.38 vs. 0.26 mean proportion of participant days that goal was achieved); and there were no significant differences during follow-up. This study shows that interventions need to experiment with designing different types of lottery schemes.

### Living for today at the expense of tomorrow

The third phenomenon is hyperbolic discounting also known as ‘present bias’ [47, 48]. Economists assume that our preferences over today versus tomorrow are the same as those over this time next week and this time in eight days. Although standard discounting simply says that we use the same discount rate in each period, evidence tells us that today looms much larger so that we discount very heavily from the present and less heavily once we think about any time into the future. So given the option, some people would choose to take £18 today rather than £20 tomorrow but would be much less inclined to take the £18 in a week’s time than £20 a day later. It has been demonstrated that the immediacy of an incentive can influence outcome of voucher-based incentive programmes for substance misuse [29], and an understanding of hyperbolic discounting should lead those designing schemes to think more carefully about when the actual incentive is given.

The immediacy of financial incentives also show potential for supporting smoking cessation in pregnancy. A review of evidence found that providing vouchers contingent on testing for smoking were effective in reducing smoking rates in late pregnancy, compared to vouchers without testing [49]. Specifically, linking the incentive to the desired outcome was clearly an important feature of the incentive design.

Similarly, an immediate financial incentive has been shown to improve adherence to anti-psychotic medications [50]. This trial offered a £15 incentive to one group of patients for each medication taken, whilst a second group received usual care. The majority of patients and clinicians felt positive about the use of incentives, and the costs were relatively low. Patients receiving the incentive were more likely to take the medication (85% vs 71%). When the incentives stopped, adherence returned to the same level as those who had not received the incentives.

### Increasing rather than decreasing payoffs

Incentives have generally been seen to be more effective for one off behaviors like vaccinations [25]. An understanding of hyperbolic discounting is particularly useful for incentive schemes where only a one-off reward is offered. For complex behaviors, multiple incentives may need to be offered at intervals but how should they be given? It has been seen that people respond more to increasing payoffs, as opposed to decreasing or constant ones [51]. This principle has been used to develop successful interventions to treat drug addiction and substance misuse and to improve medication compliance [29]. In those interventions, the patients earn points contingent on submitting urine specimens that are drug-negative or substance-negative. The reward points (incentive) usually begin at a low value and increase with each successive negative test result. If the patient fails to provide a scheduled specimen or provides a drug-positive result, then the voucher’s value is reset back to the initial low value from which it could begin to increase again. Such incentive contingency scheme can be used to improve outcomes across a wide range of different behaviors and populations.

### Reference points matter

A study from the developing world provides a further phenomenon which is that reference points matter when offering incentives. Evidence from the field, suggests that people care more about what they gain or lose around what they already have rather than what they may end up with. It is known that many people tested for human immunodeficiency viruses (HIV) in the developing world do not pick up their results. This is a major challenge to prevention campaigns and has led to a variety of ‘know your status’ campaigns. A programme in Malawi has shown that offering incentives can encourage people to pick up their HIV result [52]. The biggest increase in uptake, by around 50%, is observed when the incentive changed from zero and one-tenth of a day’s wage. Offering more money still positively affects behavior but to a much lesser degree. This finding is consistent with a ‘concave’ utility function in economics known as ‘diminishing marginal utility of income’ (more income impacts us less), but the rate at which utility declines when income rises would have to be extremely steep which is not what traditional economic models would predict. The results suggest that the utility of money is judged relative to reference points that are very contextually and narrowly defined. This finding also suggests that such locally defined reference points could influence decisions around price and the cost-effectiveness of offered incentives.

## Ethical concerns

Motivating behavior change in health is much more complex than can be accomplished with a single strategy and offering incentives (both positive and negative) are just one route to achieving improvements in health outcomes. Financial incentives are increasingly seen as an important vehicle to bring about changes in behavior that lead to healthier lifestyles and supporters and critics alike can be passionate about their use. Supporters of incentive schemes generally believe that people should be encouraged where possible into behaviors that promote improved health outcomes and that appropriately targeted incentives can reduce inequalities in health outcomes [4, 25, 53]. Incentive programmes can be seen as an example of symmetric or libertarian paternalism that steer people towards better choices without limiting what those choices are [54]. But there are also legitimate ethical concerns. Monetary compensation has the potential to lead to intrinsic motivation being ‘crowded out’ or partially destroyed [55], so that when an activity is associated with an external reward, a person may be less inclined to do the activity in the future without further rewards. However, this concern is related to the efficacy of incentives rather than the ethics. More concern comes from the perception that incentives can be seen as a form of bribery and/or coercion and inconsistent with shred social values [56].

Marteau et al. [9] suggest a psychological perspective that can help us think about the appropriateness of using incentives for encouraging participation screening tests. It is known that individuals do not always act according to their long-term goals and interests. We also know that in hindsight people often would prefer to have acted differently. For example, most of us would like to eat more healthy food, drink less, and stop smoking. Still, our behaviours do not match such intentions. Offering people a reward/incentive helps them to align their actions with such preferences. In this way, incentives enhance their autonomy to act according to their true underlying preferences. This may help explain why governments and private organisations increasingly apply financial incentives, or other extrinsic motivations, to improve health [57]. There is also evidence that the public supports incentive schemes that are cost-effective [58, 59].

Consequently, alongside further exploration of the appropriateness of using incentives in health we also need to determine whether or not they work and are cost-effective. At present we do not know what impact different incentive schemes have on various health behaviors or what impact they may have on different socioeconomic groups invited for appointments. If we learn that incentives are unsuccessful and/or are not cost-effective then more broadly implementing these interventions would be considered unwise and would not be advised. Researchers have explored the use of such incentive schemes in a range of settings, and with different populations. The results have varied, suggesting that incentives are context-dependent, and need to be planned carefully according to the needs and preferences of different groups. For example, offering small financial incentives – such as £10 in cash, or the opportunity to win £1000 in a lottery – did not improve attendance at eye screening for people with diabetes [60]. This study recruited people who had not attended their eye screening appointment in the last two years. Patients in the two incentive groups were actually less likely to attend their appointments than those who received the standard invitation. The reasons for this unexpected result were not clear, but all the patients involved were from relatively deprived groups with a history of non-attendance. The researchers also suggested that the offer of an incentive may cause a negative reaction, if the recipient believes that the screening must be unpleasant if they have to be paid to do it. This is supported by the finding that the lottery offer – to win a much larger sum – was associated with the lowest levels of attendance.

Given the promise of incentives to positively motivate behavior, we should not necessarily use legitimate concerns about their wider use in public policy prevent us from studying them in earnest. This article seeks to do this while recognising the concerns about their use. If incentives are shown to be a useful strategy to influence health behavior, a wider discussion will need to be had about the ethical dimensions of incentives before their wider implementation in different health programmes.

## Conclusions

Financial incentives are increasingly seen as an important vehicle by which to bring about changes in behavior that can lead to healthier lifestyles. Limited evidence also suggests that appropriately targeted incentives could reduce inequalities in health outcomes [53, 61]. There are lessons that can be learned from a range of disciplines and we have focused on those from behavioral economics in this article.

### Policy implications

We cannot give specific advice to policy makers for all individual circumstances, but there are some clear examples of policy and program applications of the general ideas presented in this article.

Financial incentives have been seen to be more effective in increasing performance of infrequent behaviors (e.g. vaccinations, screening) rather than in more sustained behaviors (e.g. smoking, dieting) [62]. Therefore, the incentives-related lessons from behavioral economics should be more readily applied to infrequent behaviors in the first instance. In relation to ‘overweighting small probabilities’, for example, policy makers and program designers could set up vaccinations incentive programs as lotteries, that is, ‘if you vaccinate your child, we will enter your name into a lottery, and you just might win a lot of money’.

Financial incentives for healthy behavior are already being used by large employers or health insurance providers [63, 64]. For example, patients in some states in USA have some benefits reduced or eliminated if they do not participate in health care screenings, keep their medical appointments, take their medications and adhere to health improvement programs as directed by their health care providers [65].

The United States administration also issued a rule that employers can use financial rewards and penalties for workers worth up to 50% of the health insurance premium as an incentive to quit smoking, exercise, eat healthier food, lose weight, and lower cholesterol and blood pressure [66]. In particular, the rule allows, as part employee wellness programs, employers to reward or penalize employees who meet specific standards related to their health (e.g., rewarding employees who do not use tobacco or who achieve a specific cholesterol level, weight or body mass index). In order to avoid wasting public resources, such programs will provide evidence how such incentives schemes work in the longer-term and which method is most cost-effective. Employers can improve the effectiveness of such programs by incorporating the behavioral economics principles discussed in this article.

Evidently, incentive programs directed at both providers and patients have become increasingly widespread. Although we have focused on patients and the public in this article, incentives are also frequently used to target provider behavior as a means to improve quality of care [67]. For example, in 2004 in the United Kingdom, a significant proportion of general practitioners’ pay became tied to a quality and outcomes framework [68]. The behavioral principles described here may also be applicable to incentive design on the provider side and have the potential to alleviate some of the problems identified with current programs [67]. Pay-for-performance (P4P) where providers receive financial incentives to carry out specific care or improve clinical outcomes (i.e., performance targets at which the incentive is targeted) has been widely implemented. There is emerging important evidence on P4P, which reveals a mixed picture so far. Studies in both United States [69] and United Kingdom [70] have found that initially positive effects were short lived. In diabetes care for example, the existing literature indicates P4P incentives stimulate initial gains that later tend to level off, while withdrawing the incentives partially reverts the gains [8]. We believe that behavioral re-design of some of those P4P programs is very likely to improve their effectiveness.

Finally, behavioral economics also provides some arguments against using incentives in specific circumstances. Some economists claim that offering monetary incentives may lead to destroying (‘crowding-out’) genuine (‘intrinsic’) motivation [55]. There is some evidence from a meta-analysis of experimental studies, which revealed that after a behavior is associated with an external reward, people are less willing to enact the behavior without further external rewards; in other words, extrinsic rewards undermined motivation [71]. So, when designing behavior change interventions, we must consider that badly designed policies can exacerbate the challenges we currently face and lead to unintended consequences, such as when financial incentives crowd-out/suppress intrinsic motivation for healthy behaviors [72, 73].

### Future directions

This is a debate article, so the presentation of the data is fundamentally descriptive and qualitative, because we do no aim to synthesize the evidence. Rather, we offer a theoretical investigation of incentive design and behavior change. Incentives and behavior change perhaps present the best examples of where genuine attempts have been made to span the divide between behavioral science and health policy, because incentives is the ‘tool’ most often used by the policy makers to influence behavior. However, despite the recent flurry of scientific theories and reports, there are not enough examples of successful transition from science to policy. This article aims to demonstrate that the evidence now exists where the ‘behavior incentive design’ can stimulate intervention projects informed by a scientific understanding of behavior.

This approach is useful in social sciences but is limited. For example, nudges or economic interventions that show strong effect on the collective behavior of populations are put at the same level with others that show only a tiny, and probably statistically insignificant, trend. At present, we do not have a complete enough picture of which configuration of incentives works best. So, it is worth pinpointing areas which need more research and areas where plenty of robust studies support a given recommendation.

After reviewing the mounting evidence on the uncertain effect of financial incentives to improve health behaviors, Thirumurthy, Asch and Volpp [74] conclude that the principle that individuals respond to incentives has considerable empirical support, but the devil is in the detail, because the magnitude of effects differs substantially based on the nature of the behavior, the size of the incentive, the population involved, the social context, and the design (subtle design choices in how incentives are situated, framed, or deployed can have substantial effects on their success). Therefore, investigating different ways of joining up some of the psychological mechanisms of action – loss aversion, hyperbolic discounting, and increasing payoffs – may lead to real improvements in the efficiency and effectiveness of existing and novel incentive designs. Incentives based on combinations of individual and group goals have also shown promising results. Furthermore, a potentially powerful approach is combining both patient and provider incentives, but whether this is cost-effective has yet to be determined [8].

Further insights from the behavioral sciences could allow us to combine incentive schemes with other policy tools to ensure long term effectiveness. One example are commitment devices [15], which involves individuals making a public decision about the future that results in some additional (social and/or financial) cost if they fail to follow through with that decision (or additional benefit if they successfully do so). As many people revert to past behaviors once the incentive is withdrawn, combining commitment devices with incentives may be useful in ensuring long-term behavior change. There is limited evidence from a Cochrane review that commitment contracts can potentially contribute to improving adherence to diagnostic procedures, therapeutic regimens or health promotion and illness prevention initiatives [75].

The only real way to test further interventions of this ‘joined-up’ kind is through the proper design and use of field experiments [76]. We would encourage those designing incentive schemes to consider how some of the lessons learnt from smaller scale and more experimental studies can be examined in a more naturalistic setting, thereby avoiding randomisation bias. Rigorous evaluation and dissemination of the outcomes from current and future schemes is also necessary to add to the limited evidence that tells us what does and does not work when using incentives. The ultimate criterion by which to judge the merit of such interventions is whether they improve the health and well-being of the individual; and the more is the latter, the merrier is the incentive scheme [77, 78].

## Data Availability

Not applicable
